# Luxation tibiotalienne pure: à propos d'un cas avec revu de la literature

**DOI:** 10.11604/pamj.2014.19.249.4316

**Published:** 2014-11-07

**Authors:** Mohamed Azouz, Abdel Karim Rhanim, Younes Mhamdi, Mehdi EL Alouani, Mohamed Kharmaz, Farid Ismail, Ahmed Bardouni, Abdou Lahlou, Mustapha Mahfoud, Mohamed Slaleh Berrada, Moradh Elyaacoubi

**Affiliations:** 1 Service de Chirurgie Orthopédique et de Traumatologie, CHU Ibn Sina, Rabat, Maroc

**Keywords:** Cheville, luxation, pure, articulation tibiotalienne, ankle, dislocation, pure, tibiotalar joint

## Abstract

La luxation tibiotalienne pure est une lésion extrêmement rare, causée par un traumatisme toujours de haute vélocité. Les auteurs rapportent l'observation d'une luxation tibiotalienne pure fermée chez un jeune de 19 ans, survenue à la suite d'un accident du sport (football) à déplacement postéro-médiale. La réduction a été pratiquée en urgence sous anesthésie générale. Une contention par une botte plâtrée fut assurée pendant six semaines, suivie de rééducation. Après un recul de seize mois, les résultats fonctionnels étaient excellents, sans signes d'instabilité ni d'arthrose.

## Introduction

La luxation tibiotalienne sans fracture malléolaire associée est une lésion traumatique extrêmement rare du fait de la stabilité de cette articulation, qui est fournie par la capsule et son fort complexe ligamentaire. Elle est toujours causée par un traumatisme violent et de haute énergie. Nous rapportons une luxation tibiotalienne postéro-médiale pure chez un patient de 19 ans, survenue à la suite un traumatisme de la cheville lors d'un match de football avec un revu de la littérature et les modalités thérapeutiques.

## Patient et observation

**Figure 1:**
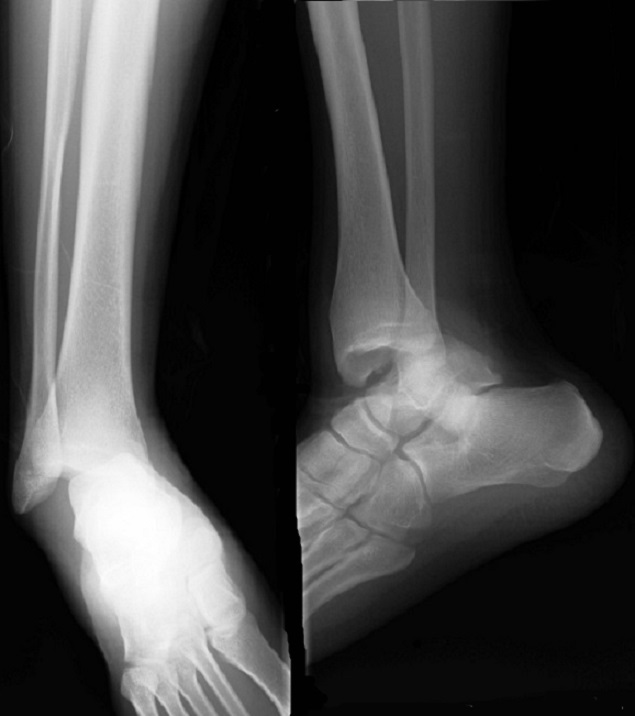
Radiographies initiales de la cheville face et profil montrant la luxation tibiotalienne pure

**Figure 2:**
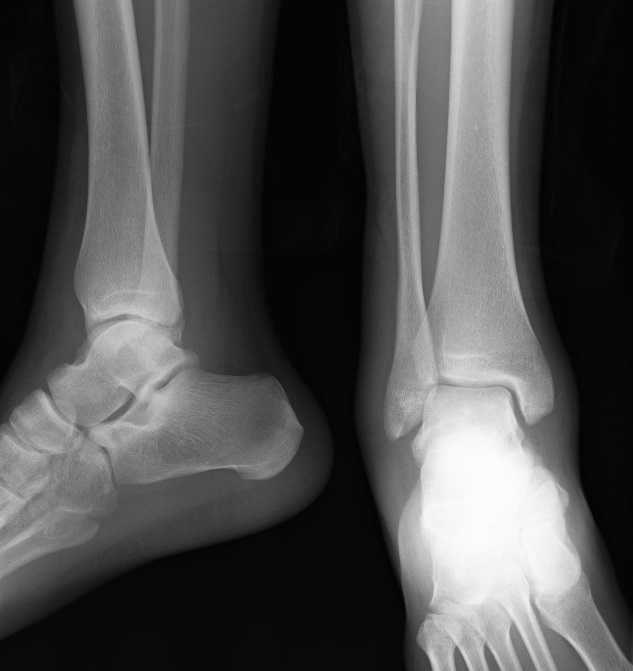
Radiographies de la cheville face et profil après réduction confirmant l'absence de fractures

**Figure 3:**
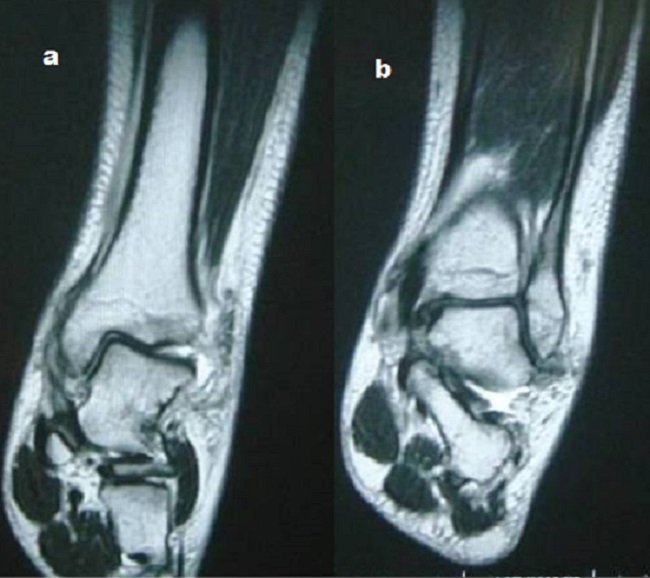
(a et b) IRM de la cheville montrant une intégrité des ligaments péri-articulaire

A Y est un jeune sportif de 19 ans sans antécédent pathologique notable, admis aux urgences suite à un traumatisme de la cheville gauche lors d'un match de football entrainant des douleurs intenses avec une impotence fonctionnelle totale du membre inférieur gauche. L'examen initiale avait objectivé une déformation de la cheville avec une douleur intenses à la palpation et à la moindre mobilisation sans déficit vasculo-nerveux ni lésion cutanée. L'examen générale n'a pas objectivé une laxité ligamentaire généralisée peut être un facteur prédisposant à ce type de blessure. La radiographie de la cheville avait objectivée une luxation tibiotalienne postéro-médiale sans fracture malléolaire associée (Figure 1) Une réduction de la luxation en urgence a été réalisée au bloc opératoire sous anesthésie générale. La radiographie de contrôle après réduction avait objectivée une bonne congruence articulaire (Figure 2). Le patient a bénéficié d'une immobilisation plâtré pendant 6 semaines suivi d'une rééducation fonctionnelle de la cheville. L'examen de la cheville réalisé après l'ablation du plâtre n'a pas retrouvé une laxité de la cheville. Une IRM de la cheville a été réalisée à la recherche d'une lésion ligamentaire avait objectivée une intégrité des ligaments péri-articulaire (Figure 3a, b). Seize mois après le traumatisme on note un très bon résultat fonctionnel avec une cheville stable et indolore et une mobilité satisfaisante.

## Discussion

La luxation tibiotalienne sans fracture malléolaire associée est une lésion très rare, souvent causée par un traumatisme de haute vélocité, jusqu'en 1995, seul 73 cas ont été rapportés dans la littérature [1]. La rareté de cette lésion peut être expliquée par la durabilité des ligaments par rapport aux malléoles et donc Lors d'un traumatisme de la cheville une fracture se produise plutôt qu'une luxation [2].

Différentes classifications ont été citées dans la littérature. En 1961, Conwell et Key ont classé la déformation en fonction de la fréquence: postérieures, antérieures, supérieures et les luxations latérales. En 1962, Kelly et Peterson ont décrit un système de classification similaire [3]. En 1965, Fahey et Murphy, en se basant sur la direction de la luxation ont cité 5 types de cette blessure: antérieure, postérieure, latérale, médiale et supérieure ou toute combinaison de ceux-ci [4]. Rios-Luna a déclaré que le type postérieur de Fahey et Murphy est la cheville dislocation la plus fréquente [5]. De nombreuses descriptions sur le mécanisme de dislocation ont été rapportées dans la littérature [3, 4]. La combinaison de la flexion plantaire et inversion forcée du pied causée par un traumatisme à haute énergie avec une charge axiale est la cause la plus fréquente de cette blessure [6]. Le talus a une forme rhomboïdale sa partie postérieure est plus étroite que sa partie antérieure. La flexion plantaire place la partie la plus étroite du talus dans la mortaise de la cheville, cette position est la plus instable. Une force d'inversion provoque alors une défaillance capsulo-ligamentaire qui provoque le risque de luxation de la cheville ou sans fracture [7]. Fahey et Murphy dans leur étude ont monté que lorsque le pied est en flexion plantaire et inversion, cela se traduit par une luxation postéro-médiale ce qui est le cas de notre patient [4].

Plusieurs facteurs de risque ont été incriminés comme prédisposant à la luxation tibiotalienne sans fracture malléolaire associée, y compris une hyperlaxité ligamentaire, une hypoplasie de la malléole interne, manque de couverture de l'astragale, la faiblesse des muscles péroniers et une histoire antérieure des entorses répétés de la cheville [2, 5, 7]. La plupart des auteurs recommandent une réduction de la luxation tibiotalienne fermée suivie d'une immobilisation plâtrée pendant 6 à 8 semaines [1, 3] mais la controverse reste pour la réparation ligamentaire en urgence. Certains auteurs recommandent la réparation des ligaments en cas de luxation ouverte [1]. La contention par une botte plâtrée sans appui pour une durée de huit semaines est adaptée de principe dans la plupart des observations [8].

La réduction en urgence est primordiale pour l'ensemble des auteurs, pour prévenir les complications comme lésions neurovasculaires, nécrose cutanée sus-jacente, les chondrolyses et la nécrose avasculaire du talus. La réduction est mieux réalisée alors que le patient est sous anesthésie générale, ce qui permet une relaxation complète des muscles [9]. Cependant dans la littérature, la réduction sanglante peut se révéler nécessaire par incarcération de la fibula derrière le tibia. Après réduction, les radiographies de stress peuvent estimer l'intégrité des ligaments de la cheville, en particulier le muscle deltoïde [8]. Pour notre patient on a opté pour une immobilisation plâtré après réduction vu le potentiel de cicatrisation par immobilisation [1].

Les résultats fonctionnels de la luxation tibiotaliennesont généralement bons avec peu de perte de l'amplitude du mouvement, parfois une enflure persistante mais des complications ont été décrites, comme l'instabilité chronique de la cheville et l'arthrose tibiotalienne [5]. Certains éléments sont de mauvais pronostic: le retard du traitement au-delà de la quatrième heure, l’état des téguments avec apparition de zones de nécrose exposant au risque d'arthrose [10].

## Conclusion

La luxation tibiotalienne pure est une lésion très rare souvent causée par un traumatisme violent dont la prise en charge en urgence adéquat est le seul garant d'un bon résultat à long terme. Notre observation illustre la place du traitement orthopédique dans la prise en charge de cette lésion.
